# Human apoA-I increases macrophage foam cell derived PLTP activity without affecting the PLTP mass

**DOI:** 10.1186/1476-511X-9-59

**Published:** 2010-06-09

**Authors:** Marius R Robciuc, Jari Metso, Anca Sima, Christian Ehnholm, Matti Jauhiainen

**Affiliations:** 1 National Institute for Health and Welfare, Public Health Genomics Research Unit and FIMM, Institute for Molecular Medicine Finland, Haartmaninkatu 8, Biomedicum, Helsinki, Finland; 2 Institute of Cellular Biology and Pathology "Nicolae Simionescu", B. P. Hasdeu 8, Bucharest, Romania

## Abstract

**Background:**

phospholipid transfer protein (PLTP) plays important roles in lipoprotein metabolism and atherosclerosis and is expressed by macrophages and macrophage foam cells (MFCs). The aim of the present study was to determine whether the major protein from HDL, apoA-I, affects PLTP derived from MFCs.

**Results:**

as cell model we used human THP-1 monocytes incubated with acetylated LDL, to generate MFC. The addition of apoA-I to the cell media increased apoE secretion from the cells, in a concentration dependent fashion, without affecting cellular apoE levels. In contrast, apoA-I had no effect on PLTP synthesis and secretion, but strongly induced the PLTP activity in the media. ApoA-I also increased phospholipid transfer activity of PLTP isolated from human plasma. This effect was dependent on apoA-I concentration but independent on apoA-I lipidation status. ApoE, ApoA-II and apoA-IV, but not immunoglobulins or bovine serum albumin, also increased PLTP activity. We also report that apoA-I protects PLTP from heat inactivation.

**Conclusion:**

apoA-I enhances the phospholipid transfer activity of PLTP secreted from macrophage foam cells without affecting the PLTP mass.

## Background

Atherosclerosis is an inflammatory disorder in the artery wall caused by the accumulation of atherogenic lipoproteins such as low density lipoproteins (LDL) and triglyceride rich remnant lipoproteins. In the artery wall these lipoproteins are modified and taken up by macrophages. This sterol loading of the macrophages promotes the formation of macrophage foam cells (MFCs) essential constituents of human atherosclerotic lesions [1]. Currently, major efforts are made to develop therapies that will promote removal of cholesterol from lesion foam cells and lead to regression of the atherosclerotic process [2]. Animal studies have shown that high density lipoproteins (HDL) can promote the removal of cholesterol from the arterial wall and transport it to the liver for excretion in a process called reverse cholesterol transport (RCT) [3]. In addition to RCT, HDL also has several other protective functions such as antioxidant, antiinflammatory, vasodilating and antithrombotic properties that contribute to the strong independent inverse relationship with atherosclerotic cardiovascular disease [3].

Phospholipid transfer protein (PLTP) is expressed by macrophages and the expression levels are increased in MFCs due to LXR activation [4-6]. Bone marrow transplantation studies in mice using PLTP deficient macrophages gave conflicting results, and this treatment either decreased [7] or increased atherosclerosis [8]. Mechanisms by which macrophage PLTP may be protective in atherogenesis involves the stabilization of ATP-binding cassette transporter A1 (ABCA1) and stimulation of cholesterol efflux [9,10], however, a recent study suggests that elevation of PLTP in macrophage does not affect RCT [11]. Furthermore, PLTP expression by macrophages results in atherogenic effects on plasma lipids and increased atherosclerotic lesion size [12].

Interestingly, it was shown that HDL levels are an important determinant of PLTP levels and it was further suggested that HDL might play a role in the stabilization of PLTP [13,14]. To date, no detailed studies addressing the effect of apoA-I on PLTP expression and secretion from human macrophages have been reported.

In the present study we have investigated the effect of exogenous human apoA-I on the synthesis and secretion of PLTP from human macrophage foam cells.

## Methods

### Cell culture, lipid loading and pharmacological treatments

Human THP-1 monocytes were purchased from the American Type Culture Collection (ATCC, Manassas, VA). The monocytes were grown and maintained in RPMI 1640 medium containing 10% (v/v) FBS, 10 mM Hepes, pH 7.4, 100 U/mL penicillin, and 100 μg/mL streptomycin at 37°C under 5% CO_2 _in a humidified incubator. To differentiate the monocytes into macrophages, the cells were plated onto 24-well plates and treated with 100 nM phorbol 12-myristate 13-acetate (PMA) (Sigma-Aldrich, St. Louis, MO) in the growth medium for 72 h prior to the experiment. The macrophages were loaded by incubating them in the presence of 25 μg of protein/well of acetylated LDL (AcLDL) in RPMI 1640 supplemented with 5% (v/v) fetal bovine lipoprotein deficient serum (LPDS), 10 mM Hepes, pH 7.4, and penicillin/streptomycin for 48 h. ApoE protein and PLTP mRNA, protein and activity levels were assessed 24 h after incubation in the presence or absence of apoA-I. The viability and attachment of the cells were carefully evaluated by light microscopy and protein measurements and no cytotoxic effects could be observed.

### SDS-PAGE and Immunoblot Analysis

Equivalent amounts of protein from the cell lysate and medium were subjected to Western blot analysis. Medium was concentrated 50-fold for the detection of apoE and PLTP. To detect apoE we used a monoclonal antibody raised against the human apoE or a horseradish peroxidase (HRP)-conjugated polyclonal antibody specific for human apoE (DAKO, Denmark). For PLTP detection we used a mouse monoclonal antibody (Mab59) or a rabbit polyclonal antibody raised against purified human plasma PLTP. Cellular actin was detected using a specific rabbit polyclonal antibody (Santa Cruz).

### ApoE ELISA

Human apoE was detected by ELISA as described previously [15] with some modifications. Briefly, we used a polyclonal rabbit antibody (R107) as a capture antibody to coat 96-well plates. As a detection antibody we used a HRP-conjugated polyclonal antibody specific for human apoE (DAKO, Denmark). Standard curve was prepared using a standardized serum (Daichi, Japan).

### Gene expression analyses

PLTP mRNA levels were measured by real time PCR using as forward primer 5'-ACGCAGGGACGGTCCTGCTC-3' and as reverse primer 5'-CTCATTGAGCATGGGCATCACCCC-3' [5]. Cultured cells were homogenized in RLT buffer (Qiagen, Valencia, CA) and total RNA was isolated with RNeasy Mini kit (Qiagen) according to the manufacturer's instructions. The total RNA (2 μg) was reverse-transcribed by using Superscript II (Invitrogen, Carlsbad, CA) and random hexamer primers (Applied Biosystems, Foster City, CA). Samples were amplified in triplicate for PLTP and two housekeeping genes, GAPDH and 18S rRNA, on a 7000 Sequence Detection System (Applied Biosystems), using a SYBR-green kit (Applied Biosystems).

### Purification of PLTP from human plasma

PLTP was purified from human plasma according to Marques-Vidal *et al*. [16]. Briefly, the human plasma fractions, eluted from Butyl-Toyopearl 650(M) column, containing PLTP activity were combined and applied to a first heparin-sepharose column. After extensive washing with the equilibration buffer, PLTP activity was eluted with 25 mM Tris-HCl buffer, pH 7.40, containing 1 mM EDTA and 1.0 M NaCl. The fractions containing PLTP activity were combined, dialysed against 25 mM Tris-HCl buffer, pH = 7.40, containing 1 mM EDTA, and applied to a Mono Q HR 5/5 column, attached to a Merck HPLC system. The column was eluted with a linear NaCl gradient (0-0.5 M) prepared in equilibration buffer. The fractions containing PLTP activity were combined, dialysed against 25 mM Tris-HCl buffer, pH 7.40, containing 1 mM EDTA and then applied on a second heparin-sepharose column (Hi-Trap, 1 ml total volume, Pharmacia, Upsala, Sweden). The column was then eluted with a linear NaCl gradient (0-0.5 M). Active fractions from the Hi-Trap column were pooled and applied to a hydroxylapatite column (2 ml total volume) previously equilibrated with 1 mM Na-phosphate buffer, pH 6.80, containing 150 mM NaCl. PLTP was eluted with a linear Na-phosphate gradient (1-500 mM). PLTP activity eluted at a phosphate concentration of 125-150 mM. PTLP activity ranged between 2000 and 6000 nmol phospholipid transferred/h/ml depending on the preparation and was devoid of H-TGL, LCAT, phospholipase and CETP activity.

### PLTP activity

PLTP activity was measured using a radiometric method as previously described [17]. Cholesterol loaded macrophage media was 5-fold concentrated by ultrafiltration and washed twice with PLTP activity assay buffer before the activity was measured.

### ApoA-I lipidation

For apoA-I lipidation we used increasing amounts of L-α-Phosphatidylcholine (Sigma-Aldrich, St. Louis, MO). The molar ratio of the proteoliposomes, apoA-I:PC:CHOL, was as follow: 1:50:0, 1:50:7, 1:100:0, 1:100:7, 1:150:0 and 1:150:7. PC and CHOL were dried under nitrogen at room temperature in glass tubes and resuspended in the assay buffer (10 mM Tris-HCl, 1 mM EDTA, 140 mM NaCl, pH 7.4). ApoA-I was added to the mixture at a concentration of 1 mg/ml. After the addition of Na-cholate the samples were mixed gently avoiding foaming followed by 20 minutes incubation at 24°C in a water bath with gentle shaking. Finally the samples were dialyzed against assay buffer for 20-40 hours and stored at + 4°C.

## Results

**Figure 1:**
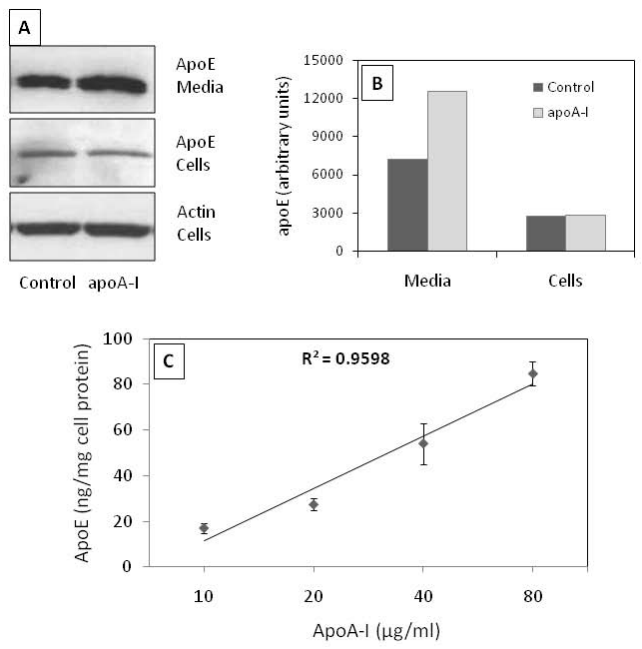
**Induction of apoE secretion by apoA-I in macrophage foam cells (MFCs)**. **Panel A**, apoE was analyzed by Western blot in the media and cellular lysate from lipid loaded macrophages incubated in the presence of 10 μg/ml apoA-I (apoA-I) or without (Control) for 24h. **Panel B, **bands were quantified (ImageJ v1.42q software) and the bars represent the intensity of apoE bands normalized to intensity of actin band. **Panel C**, increasing concentrations of apoA-I were added in the culture media of MFC and apoE concentration was quantified after 3h of incubation. ApoE concentration was determined by ELISA and values were normalized to total cell protein concentration. A linear relationship between apoA-I and apoE concentration was observed (R^2 ^= 0.959). The dots and error bars represent the mean ± SD calculated from triplicates. The results are representative for at least 3 independent experiments.

Loading of THP-1 macrophages with AcLDL resulted in an increase in apoE, PLTP and cholesteryl-ester transfer protein (CETP) secretion (data not shown). When ApoA-I was added to the media of cholesterol loaded macrophages (Figure 1) the apoE secretion was increased. ApoA-I addition did not affect the cellular content of apoE (Figure 1, panel A and B). ApoE secretion from the cells was measured by a specific ELISA in serum free media containing 10, 20, 40 and 80 μg/ml apoA-I and a concentration dependent induction in the secretion (up to 5-fold) could be observed (Figure 1, panel C).

**Figure 2:**
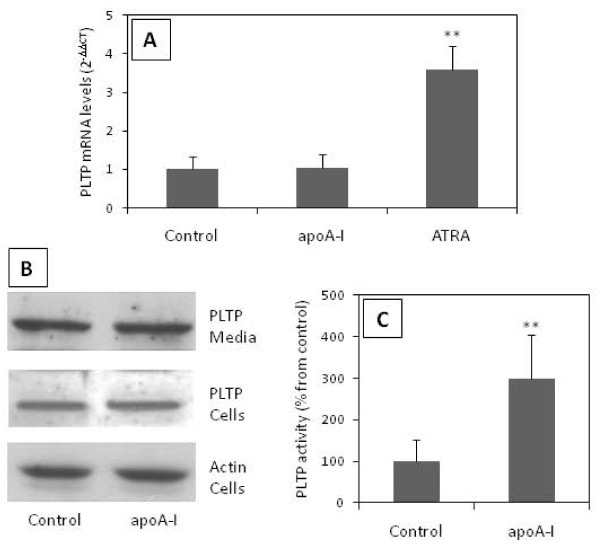
**Effect of apoA-I on PLTP derived from macrophage foam cells**. **Panel A**, PLTP gene expression was analyzed by real-time PCR in untreated cells (Control) and cells treated with 10 μg/ml apoA-I for 24 h (apoA-I). As a positive control induction of PLTP gene expression was measured after all-trans retinoic acid (ATRA) addition. To normalize the data we used as control two housekeeping genes, GAPDH and 18s rRNA. **Panel B**, PLTP was detected by Western blot in media and cell lysates from cells incubated in the presence (10 μg/ml) or in the absence (Control) of apoA-I. Actin was used as a reference. **Panel C**, PLTP activity measured in media from cells incubated in the presence (10 μg/ml) or in the absence (Control) of apoA-I in the media. Bands represent the mean ± SD calculated from triplicates. Figures are representative for at least two independent experiments. ** P < 0.01.

The stimulating effect of apoA-I on apoE secretion from macrophages has been reported by several groups and also confirmed by us in the present study. We further investigated whether apoA-I also affects PLTP secretion from macrophages, as both, PLTP and apoE, are known to be important players in macrophage cholesterol homeostasis and are associated with HDL or apoA-I [18]. Moreover, PLTP deletion from macrophages reduces apoE secretion [19] suggesting that PLTP could play a role in the apoA-I induced apoE secretion. Real time PCR quantification showed no effect of apoA-I on PLTP mRNA levels whereas all-trans retinoic acid (ATRA), agonist for Retinoic X Receptor (RXR), induced a 3.5-fold increase in the relative PLTP mRNA expression (Figure 2, panel A). Western blot analysis revealed that, there was no apoA-I mediated effect on cellular levels of PLTP and, in contrast to apoE, also in the media PLTP levels remained similar after the apoA-I addition (Figure 2, panel B). In contrast, addition of an LXR agonist (22-OH cholesterol) markedly increased PLTP secretion from the cells (data not shown). PLTP activity in the media was 3-fold increased by the addition of 10 μg/ml apoA-I, although apoA-I had no effect on PLTP protein levels in cell culture media (Figure 2, panel C).

**Figure 3:**
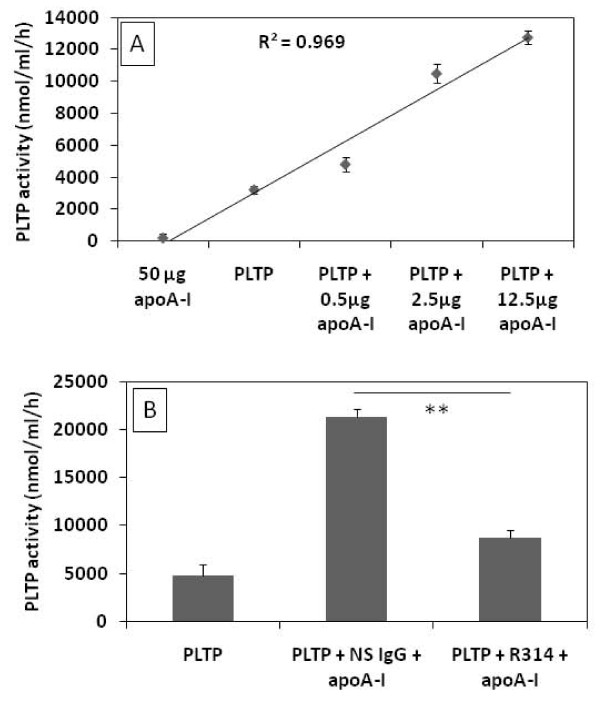
**Effect of apoA-I on the activity human purified PLTP**. **Panel A**, PLTP isolated from human plasma was pre-incubated for 2 hours at room temperature in the presence of increasing concentrations of apoA-I and thereafter assayed for phospholipid transfer activity. As a control, 50 μg apoA-I were added to the reaction mixture in the absence of PLTP. A linear relationship between apoA-I concentration and PLTP activity was observed (R^2 ^= 0.969). Dots and error bars represent mean ± SD calculated from triplicates. **Panel B**, apoA-I was pre-incubated with non-specific rabbit polyclonal antibodies (NS IgG) and anti-apoA-I rabbit polyclonal antibodies (R314) and the effects on PLTP activity were evaluated. Figures are representative for three independent experiments. Bars and error bars represent mean ± SD calculated from triplicates. **P < 0.01.

Because the macrophage media also contains apoE that presumably could mediate the apoA-I induction of PLTP activity we also performed experiments where only purified human plasma PLTP and lipid free apoA-I were co-incubated. ApoA-I significantly induced purified PLTP activity in a concentration dependent manner (Figure 3, panel A). To exclude the possibility that the effect observed on PLTP activity could be due to the addition of lipid free apoA-I in the PLTP assay reaction mixture, we evaluated the phospholipid transfer of apoA-I in the absence of PLTP. No phospholipid transfer could be detected with 50 μg apoA-I, 4-fold higher than the highest amount of apoA-I used with PLTP (Figure 3, panel A). The apoA-I mediated induction of purified PLTP activity was abolished by a specific anti-apoA-I polyclonal antibody whereas the non-specific antibody had no effect on the activity (Figure 3, panel B).

**Figure 4:**
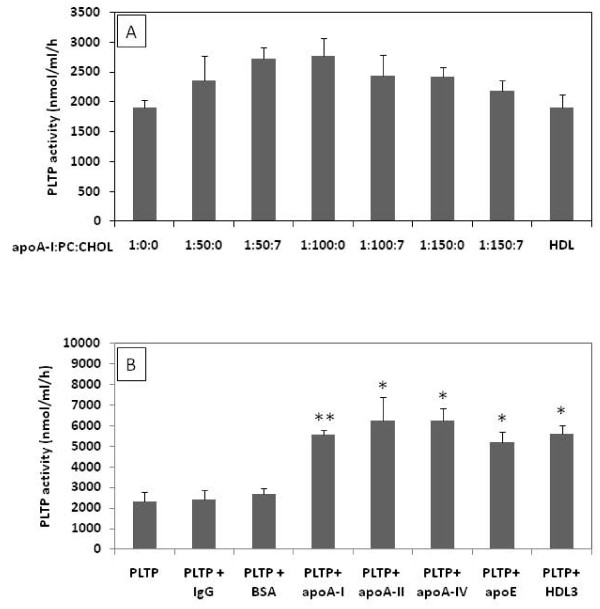
**The capacity of lipidated apoA-I and other apolipoproteins to induce PLTP activity**. **Panel A**, PLTP was incubated for 2 hours at room temperature in the presence of 10 μg/ml of lipid free apoA-I or lipidated with increasing amounts of phosphatidylcholine (PC) and cholesterol (CHOL) and 10 μg/ml HDL (representing a highly lipidated form of apoA-I) and assayed for the phospholipid transfer activity. **Panel B**, PLTP was incubated for 2 hours at room temperature in the presence of 10 μg/ml of purified rabbit imunoglobulins (IgG), bovine serum albumin (BSA), apoA-I, apoA-II, apoA-IV, apoE and HDL and analyzed for phospholipid transfer activity. Figures are representative for three independent experiments. Bars and error bars represent mean ± SD calculated from triplicates. * P < 0.05, **P < 0.01

We have further questioned whether the observed effect of apoA-I on PLTP activity depends on the apoA-I lipidation or whether it is restricted to apoA-I only. To answer the first question, we have lipidated apoA-I with increasing amounts of PC, as described in material and methods. As depicted in figure 4 (panel A), increased apoA-I lipidation did not have a significant effect on the observed induction of PLTP activity. In addition, HDL isolated from human plasma was equally effective in inducing the PLTP activity. Furthermore, we also showed that lipid-free apoA-II, apoA-IV and apoE induced purified PLTP activity to a similar extent as apoA-I (Figure 4, panel B). In contrast, proteins not belonging to the apolipoprotein family of proteins such as rabbit non-specific immunoglobulins and bovine serum albumin had no significant effect on PLTP activity (Figure 4, panel B).

**Figure 5:**
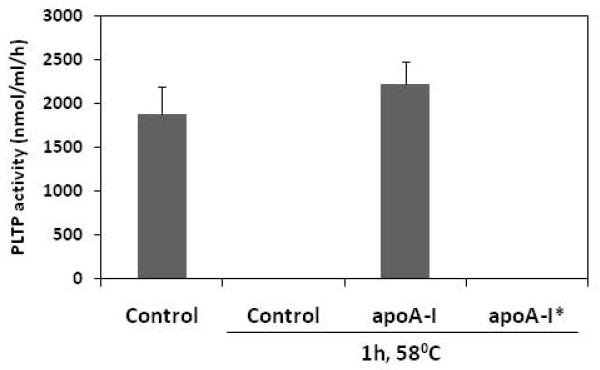
**Stabilizing effect of apoA-I on PLTP activity**. PLTP was incubated at 58°C for 1 hour in the presence or absence of 20 μg apoA-I and assayed for the phospholipid transfer activity. Figure is representative for two independent experiments performed with two different PLTP batches. Bars represent mean ± SD calculated from duplicates. *20 μg apoA-I was added to PLTP after the incubation at 58°C for 1 hour.

Plasma purified PLTP can be inactivated by heating at 56°C for one hour (Figure 5) [20]. To test if apoA-I can protect PLTP from heat inactivation, we preincubated purified PLTP from plasma with 20 μg apoA-I. Preincubation of PLTP with apoA-I preserved the phospholipid transfer activity, but the addition of apoA-I after heat inactivation of PLTP failed to recover the lost phospholipid transfer activity (Figure 5). This shows that apoA-I not only enhanced phospholipid transfer protein activity of PLTP but has also a stabilizing effect.

**Figure 6:**
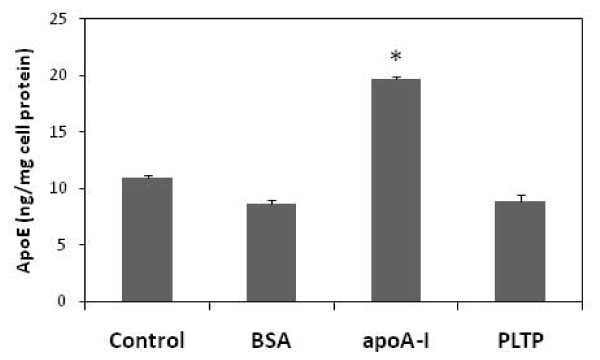
**Effect of PLTP on apoE secretion**. ApoE was analyzed by ELISA in the media from MFC incubated with 10 μg/ml bovine serum albumin (BSA), 10 μg/ml apoA-I (apoA-I) or with 200 nmol/ml/h PLTP for 24h. Figure is representative for two independent experiments. Bars represent mean ± SD calculated from triplicates. * P < 0.05.

Finally, because apoA-I enhances both PLTP activity and apoE secretion from MFC we questioned whether apoA-I mediate its effect on apoE secretion by enhancing the PLTP activity. To test this we added plasma purified PLTP in the media of MFC and evaluated the apoE secretion using ELISA. As depicted in figure 6, addition of PLTP (200 nmol/ml/h) was equally ineffective in stimulating apoE secretion from MFC as the addition of bovine serum albumin.

## Discussions

PLTP synthesized by macrophage and MFCs has been associated with the development of atherosclerosis. In the current study we investigated the effect of apoA-I on PLTP secreted from MFC.

Our results confirm existing data which show that apoA-I strongly stimulates apoE secretion from macrophages, an effect that is likely to contribute to the antiatherogenic potential of HDL. Interestingly, the enhancement of apoE secretion induced by apoA-I is concentration dependent, but even at levels as high as 80 μg/ml a saturation of the system could not be demonstrated. This suggests that the system capacity is very high and can have important consequences in MFCs physiology as well as in plasma lipoprotein metabolism.

PLTP secreted by macrophages contributes significantly to total plasma phospholipid transfer activity [7]. The plasma PLTP pool derived from macrophages has most probably a major impact on plasma lipoprotein metabolism and atherogenesis. In the present study we demonstrate that apoA-I does not affect PLTP secretion from macrophages but instead increases PLTP facilitated phospholipid transfer activity. The induction of phospholipid transfer is due to a direct effect of apoA-I on PLTP since the incubation of purified PLTP in the presence of apoA-I resulted in an effect similar to that observed with macrophage derived PLTP. These *in vitro *observations suggest that this effect most probably is valid for PLTP secreted from other tissues. Indeed, the PLTP activity in ABCA1 deficiency, that results in very low levels of apoA-I and HDL in plasma, is significantly reduced [13,14]. The effect we report here is specific not only for apoA-I since an increase of PLTP activity was also observed following incubation in the presence of other apolipoproteins such as apoA-II, apoA-IV and apoE. This suggests that PLTP requires interactions with proteins containing amphipathic α-helix domains for proper lipid transfer activity. Interestingly, similar structural characteristics are needed for the induction of apoE secretion from macrophages [21].

It is well known that PLTP is associated in plasma with HDL [22] and the particles containing PLTP are highly variable regarding the phospholipid transfer capacity [23]. It is not clear yet what is the determining factor for the differences in the PLTP activity and this remains to be established [24,25]. Although the interaction of PLTP with lipoprotein surfaces is an obligatory component of lipid transfer, so far, it was not clearly demonstrated that apoA-I and HDL can actually enhance PLTP activity.

It was recently reported that macrophage PLTP deficiency causes a significant reduction of apoE secretion *in vivo *[19]. The mechanism behind this observation is not known but it may involve the stabilizing effect of PLTP on ABCA1, known to be responsible for basal apoE secretion [21]. A second possibility is that PLTP could mediate HDL induced secretion of apoE from macrophages. Our observations, that apoA-I does not affect the synthesis and secretion of PLTP from macrophages and that increased PLTP activity in macrophage media, by addition of exogenous active PLTP, do not increase apoE secretion from macrophages, are indirect evidence that the first hypothesis is more likely.

## Conclusions

In summary, we have demonstrated that, in cholesterol loaded macrophages, apoA-I does not affect PLTP synthesis or secretion but increases PLTP mediated phospholipid transfer activity. The same was observed with purified plasma PLTP and the effect was independent on apoA-I lipidation status and could be demonstrated by other proteins containing amphipathic α-helix domains. We also provide evidence that the apoA-I induced apoE secretion from macrophages and the enhancement of PLTP activity by apoA-I are unrelated phenomena.

## Competing interests

The authors declare that they have no competing interests.

## Authors' contributions

**MRR **conceived and designed the study, carried out the majority of the experiments and wrote the manuscript. **JM**, carried out PLTP activity assays and help with the experimental design for *in vitro *studies. **AS**, participated in the study design and coordination, and revised the manuscript for important intellectual content. **CE**, participated in the design of the study and revised the manuscript for important intellectual content. **MJ**, coordinated the study, made substantial contributions to its conception and design and revised the manuscript for important intellectual content.

All authors read and approved the manuscript.
